# Dynamic Systems Approach in Sensorimotor Synchronization: Adaptation to Tempo Step-Change

**DOI:** 10.3389/fphys.2021.667859

**Published:** 2021-06-21

**Authors:** Nima Darabi, U. Peter Svensson

**Affiliations:** Department of Electronic Systems, Norwegian University of Science and Technology, Trondheim, Norway

**Keywords:** sensorimotor synchronization, period correction, rhythmic perception, system identification, root locus analysis, pole/zero systems, frequency domain, tempo step-change

## Abstract

This paper presents a dynamic systems model of a sensorimotor synchronization (SMS) task. An SMS task typically gives temporally discrete human responses to some temporally discrete stimuli. Here, a dynamic systems modeling approach is applied after converting the discrete events to regularly sampled time signals. To collect data for model parameter fitting, a previously published pilot study was expanded. Three human participants took part in an experiment: to tap a finger on a keyboard, following a metronome which changed tempo in steps. System identification was used to estimate the transfer function that represented the relationship between the stimulus and the step response signals, assuming a separate linear, time-invariant system for each tempo step. Different versions of model complexity were investigated. As a minimum, a second-order linear system with delay, two poles, and one zero was needed to model the most important features of the tempo step response by humans, while an additional third pole could give a somewhat better fit to the response data. The modeling results revealed the behavior of the system in two distinct regimes: tempo steps below and above the conscious awareness of tempo change, i.e., around 12% of the base tempo. For the tempo steps above this value, model parameters were derived as linear functions of step size for the group of three participants. The results were interpreted in the light of known facts from other fields like SMS, psychoacoustics and behavioral neuroscience.

## Introduction

Sensorimotor synchronization (SMS) is defined as the coordination of rhythmic movement (motor) with an external rhythm (sensory) (Repp, 2005; Repp and Su, 2013). Such rhythmic coordination of perception and action, or the rhythmic synchronization between a timed sensory stimulus and a motor response (Michon and Van der Valk, 1967), is often studied in the context of rhythmic perception and collaboration, especially music performance.

One standard experiment in the study of SMS deals with the task of synchronizing an action to a temporally regular (isochronous) series of impulses and has applications beyond music co-performance, such as in dance (Miura et al., 2011) or computer gaming (Bégel et al., 2017). When reducing the input (the sensory stimulus) to a metronome, i.e., an isochronous sequence of tones or clicks, the action is often reduced to a simple task of hand clapping or finger tapping in synchronization with the input (Repp, 2005). A variety of models have been used to study the relationship between such input and output. These models typically rely on a simple assumption that, based on observations of previous beat timings, the participant can predict the timing of the next beat and make anticipatory actions, for example aiming at being synchronous to it. In this paper, we are bringing models from dynamical systems theory and control systems engineering to the study of SMS and applying them on data from a task of synchronization to a suddenly changing metronome tempo. The primary goal is to apply dynamic systems modeling to the inherently discrete-time system of an SMS task. The use of a separate linear, time-invariant system for each tempo step is a limitation, but model parameter trends might be developed into more advanced non-linear models, which could be applied to more general stimulus signals.

### History

Studies on rhythm and perception started in the early years of experimental psychology with pioneer works by Stevens (1886) and Wilhelm Wundt back in the 1890s [cited in Blumenthal (1975)]. Early work included experimental studies on the speed of synchronization with an external rhythm (Nicholson, 1925) [cited in Delignières et al. (2004)] and properties of asynchrony in adaptation to different tempos, which led to the definition of upper limits of the human rates (Woodrow, 1932). Later, notable contributions were made in the 1950s and 1960s through the works of Paul Fraisse [cited in Repp (2005)] and Michon and Van der Valk (1967). Michon studied the response to rhythmic perturbations including step changes, ramps and sinusoidals and sums of sinusoidals. He also made the first attempt at formulating a standard set of descriptive terms to describe research on SMS. In later years Bruno H. Repp (Repp and Keller, 2004; Repp, 2010) and Jeff Pressing (Pressing and Jolley-Rogers, 1997; Pressing, 1998, 1999) presented several important findings, including two extensive overview studies (Repp, 2005; Repp and Su, 2013).

### Study of Sudden Step Changes

One particular task, which is common in the studies of SMS, is to respond to sudden tempo changes. Michon used inter-onset intervals (IOIs) (the time between two consecutive clicks) of 600, 1200, and 2400 ms and step changes of 8, 16, and 32% of the base value, and one step up and one step down for each test on five subjects (Michon and Van der Valk, 1967). The results revealed an initial overshoot in the rate of the response for increasing tempo changes, as well as an undershoot for decreasing ones, generally within 4–5 taps. He proposed a linear predictor model that could account for 70% of the data collected from three of his five subjects. Parameterizing the experiment and identifying the parameters he spotted some non-linearity; that the quality of performance depended on the step size and the baseline IOI. However, he did not elaborate on the nature of this dependency nor included a higher resolution of tempo changes or a variety of tempo baselines.

Later studies started to make a difference between the subliminal step changes that are below the threshold for conscious perception, and the supraliminal changes that are above that threshold (Thaut M. et al., 1998; Thaut and Kenyon, 2003). Mates (1994) further defined two hypothetical internal processes called phase and period error correction processes that will be briefly reviewed in section “Event and Interval Variables and the Choice Between the Period Error Correction and Phase Correction Process” due to their relevance to the stages of data preparation in this work. He also made a distinction between internal processes and external timing and attempted to explain the often observed overshoot for step responses and showed that period correction is slower for undetected than for detected changes, even when they were of the same magnitude (Repp,2001a,b), implying that phase correction is rapid and automatic, whereas period correction can be dependent on awareness of a tempo change (Repp,2001a,b) (Hary and Moore, 1985) reported adaptation to subliminal step changes to be very slow and gradual. Thaut M. H. et al. (1998) investigated the adaptation by five subjects to an unexpected step change of 2, 4, or 10%, for an input interval of 500 ms in a finger-tapping task and reported a strategy shift based on the percentage of the introduced step change. They also reported a relatively rapid adaptation to both large and small step changes, though the overshoot occurred only after large, detectable changes. Repp and Keller (2004) observed the suppression of period correction, but not phase correction, when asking participants to continue tapping at the initial tempo and ignoring the step change. They concluded that phase correction is a lower-level cognitive process, whereas period correction could have higher-level cognitive components. Repp (2002) also considered phase correction to be purely specific to SMS tasks whereas period correction could be more related to expressing timing in music. It is reported that over-correction above the threshold of awareness happens as a result of participants adjusting the timing of their taps by a larger amount than would be necessary to compensate for the full asynchrony (Van Der Steen and Keller, 2013). The observation of overshoot has also been reported for continuous, rather than sudden, step-changes. Schulze observed a systematic alternation of under-adjustment and over-adjustment of period and phase correction in synchronization with a metronome that smoothly changed tempo, from slow to fast (accelerando) or from fast to slow (ritardando) (Schulze and Vorberg, 2002). For experiments combining step changes with phase see Large et al. (2002), and with antiphase tapping, see Thaut and Kenyon (2003).

Fraisse and Repp (2012) reported a situation in which participants began to synchronize with a sequence when its tempo was not known in advance and observed that about three taps were needed to tune in to a sequence if the tapping started immediately after the first tone. From the third stimulus (or the third cycle in the case of a simple repeated rhythm) onward, simultaneity was achieved with an error of less than 50 ms.

### Discrete and Continuous Approaches to SMS

In studies of SMS, there are two main theoretical approaches: information processing and dynamic systems theory (Torre and Balasubramaniam, 2009). Information-processing theory describes the rhythmic responses and stimuli as event-based discrete time series and aims at describing hypothetical internal processes underlying the behavior (Repp, 2005). Dynamic systems theory approaches, on the other hand, is less focused on the inner-workings of the systems, usually takes a black-box approach, and is concerned with the mathematical description of the observable synergies. Given that the focus of dynamic systems theory is on continuous, non-linear, and within-cycle coupling (Large, 2008), it has typically been used for continuous movement tasks, such as circle drawing in sync with external stimuli (Todd et al., 2002; Repp, 2005). Comparing the results obtained from series of time intervals produced in discrete finger-tapping tasks with the spectral analysis of synchronization-continuation experiment reveals that movements that are organized as a series of discrete contacts are consistent with an event-based timing model and require more explicit temporal control than continuous movements such as the oscillatory motion of the hand (Delignières et al., 2004). Although dynamic systems theories are general enough to encompass both continuous and discrete forms of periodic movements, they have primarily been used to analyze continuous movement tasks (Repp, 2005). Moreover, when the temporal goal is defined externally (e.g., by a metronome), timing initially requires an event-based representation but after the first few movement cycles, control processes become established that allow timing to become emergent and continuous (Ivry et al., 2002; Zelaznik et al., 2005). It is also shown that event-based and emergent timing can coexist in a dual-task of discrete (rhythmic tapping) and continuous (circle drawing) (Repp and Steinman, 2010). These observations imply that both continuous and discrete processes might be involved in discrete finger-tapping tasks and point to the potential suitability of dynamic modeling in tasks of discrete finger-tapping.

A central hypothetical notion in the dynamic modeling of SMS is the “internal timekeeper” that keeps track of time intervals of the perceived rhythm of a metronome, other musicians, or a self-paced rhythm. Many attempts at modeling SMS behavior use this notion as a basis for models of phase or period error correction processes by assuming a quantifiable correction of the timekeeper interval that ultimately affects the generated output (Michon and Van der Valk, 1967; Wing and Kristofferson, 1973; Mates, 1994; Thaut M. H. et al., 1998). Michon and Van der Valk (1967) introduced regularly time-sampled analysis to SMS and dynamic modeling of rhythmic behavior. He proposed a time-order representation that transforms the data from an irregularly sampled format to a regular time series, enabling discrete-time analysis that is otherwise inapplicable. Such representation has since been used by those taking an information-processing approach to the dynamic modeling of rhythmic behavior (Wing and Kristofferson, 1973; Mates, 1994).

### Approach in the Present Study

As reviewed in section “Study of Sudden Step Changes,” literature on SMS research tends to describe the qualitative difference between subliminal and supraliminal step-changes in terms of the existence of, or the magnitude of, an overshoot in the output intervals. Furthermore, the time of adaptation has been used to describe this difference, either expressed directly as the time, or in terms of the number of tap/tones that it takes for adaptation to take place. Such a tendency reflects a tradition of studying the signals from SMS experiments merely in the time domain. The existence or the relative size of such an overshoot, as well as the time of adaptation, can be better formulated by a new set of parameters that are described in the frequency domain. This study will hence present a model that introduces a new set of parameters, which leads to a better understanding of the behavior. The new parametrization uses parameters such as frequency of oscillation and damping ratio and can account for such observable qualities in the time signals. To do so, we introduce system identification as a new approach to model SMS as a dynamic system. The simple scenario here is demonstrated to study the response of the “system,” a human in an SMS task, to a stimulus with a step-wise changing tempo.

As introduced above, we are dealing with a discrete task of generating rhythmic impulses, but we take a continuous-time approach as in dynamic systems theory. The difficulty in such an approach to SMS, as reported by Michon and Van der Valk (1967), is that the discrete nature of typical tapping/clapping experiments obstructs the underlying continuous process from manifestation. The goal is to determine such a continuous process, while the task is discrete, and the inter-sample behavior of the system is not accessible in our limited experimental setup. The behavior of a human subject is assessed merely at its input/output level and sampled only at the time of performed onsets. We do not have access to intermediate data related to the behavior of the system between the two onsets, such as electrophysiological monitoring of the brain activity, brain imagery, or any other behavioral data collected from the subjects at a higher sampling frequency than the frequency of the rhythmic SMS task. In this context, the term inter-sample behavior relies on the assumption that there is a continuous internal process, which is reacting to discrete events (Wing and Kristofferson, 1973).

To overcome the problem of unknown inter-sample behavior, we will first upsample the sampled data collected at the input/output level of the human subject such that the temporal signals of pulses can be viewed as continuous-time signals in this analysis^1^ We will then use a dynamic systems approach with a tool which, to our best knowledge, has not been applied in the study of SMS: so-called system identification, which is a standard tool in cybernetics, control theory and systems theory.

In control theory, signal processing, and cybernetics, *state-space models* mathematically describe a physical system^2^ by a set of input, output and state variables. These models can be non-linear if they do not satisfy the properties of superposition^3^. They can also be time-variant, i.e., the state variables of the model can change over time. We will show in the result section “Event and Interval Variables and the Choice Between the Period Error Correction and Phase Correction Process” that the system which we are trying to model shows non-linearity, e.g., halving the step size of the input would not cause a halved output but will instead change the behavior of the system. Therefore, our model parameters can depend on the size of the step-change.

The non-linear behavior of humans can be attributed to the finite thresholds of perceptual systems, as well as the human ability to learn and adapt to situations. This is because the human tracker is more adaptive than a fixed linear system and takes advantage of the predictability in a tracking task. Additionally, humans may not respond based on moment-to-moment input, as expected from a linear system, but would instead detect and respond to patterns when they are present (Jagacinski and Flach, 2018). This makes the identification of a fixed human transfer function impossible. Non-linear models could have been used to identify a control system vary, and many such possibilities exist (Cloosterman et al., 2010; Strogatz, 2018).

However, as we will discuss in section “Results,” it is possible to view this particular non-linear system as a system that behaves as a linear, time-invariant system (LTI) for one particular input signal. The system parameters will change gradually when the input signal changes gradually. This reduction, however, usually requires cutting down some dimensions of the data. In this paper, we will model each participant at a fixed tempo step with an LTI system using the *System Identification Toolbox^TM^* in MATLAB^©^. This toolbox uses an iterative prediction-error minimization method to update the initial model parameters to fit the given input and output data in a discrete-time state-space model. Using this method, we can identify the relationship between the input and output of a linear time-invariant single-input single-output (SISO) system.

Due to the noisy character of the data, quite a large number of variations and repetitions were used, and this scale, together with the exploratory scope, limited the feasibility of a larger-scale experiment. To get enough experimental data to use with this method, then, a series of tests with finger-tapping tasks in response to stepwise tempo changes for isochronous clicks as stimuli were run with three participants.

### Event and Interval Variables and the Choice Between the Period Error Correction and Phase Correction Process

Consider a one-to-one task for a person who tries to synchronize responses *R*_*j*_ to a sequence of stimulus pulses *S*_*j*_. Mates referred to the time instants of stimulus and response events as “event” variables (“reading of a clock”), and used capital letters (Mates, 1994). Temporal differences between two event variables were then used for creating a new, derived set of data-points: *interval* variables, symbolized by lower-case letters. For example, the difference between two adjacent elements of *R*_*j*_, is known as *inter-response interval* or IRI, denoted by *r*_*j*_ = *R*_*j*_−*R*_*j*−1_. Similarly, *s*_*j*_ = *S*_*j*_−*S*_*j*−1_ is another interval variable called *inter-stimulus interval* or ISI. It is also common in the SMS literature to use the term IOI to refer to either ISI or IRI, an interval variable whether it is from the stimulus or the response.

In the SMS literature, two error correction processes are thought to be underlying auditory sensorimotor behavior. For the successful accomplishment of a SMS task, both types of event and interval variables need to be in sync, as perceived by the person. In other words, the successful production of a sequence of motor acts in synchrony with a rhythmic sequence of stimuli, requires both synchronization of time events and minimization of the discrepancy between the time intervals. Two internal error correction processes are usually defined in the studies of SMS corresponding to the two mentioned coordination tasks (Mates, 1994).

A *phase error correction process* deals with the correction of synchronization error (or *asynchrony*), the time difference between the input stimuli and output response, *R*_*ij*_−*S*_*ij*_. A *period error correction process*, on the other hand, tries to correct the mismatch between the input and output intervals, IRI and ISI, for example to minimize the value of *r*_*j*_−*s*_*j*_ which is also known as *discrepancy*. When the data is collected only at a final input/output endpoint, these processes are not uniquely identifiable or separable from each other. We cannot tell to which extent each process has contributed to the shifting of the timestamp of a performed onset. Moreover, simulations have shown that their identified parameters can be highly interdependent (Schulze and Vorberg, 2002). Therefore, many studies have attempted to isolate only period correction by using step changes, or phase correction by using phase perturbations (Repp, 2005; Repp and Su, 2013).

The phase error correction process deals with precision in timing, while period error correction is in charge of the rhythm stability. There is a logical assumption that in a rhythmic SMS task, the latter is prior to the former, i.e., in the absence of a stable rhythm during an SMS task, subjects would not prioritize timing accuracy. The adjustment of the interval variables (coping IRI with ISI) is necessary for the synchronization of the event variables. Mathematically speaking, in the presence of a considerable discrepancy where interval variables are not in sync, temporal synchronization between the event variables will be an accidental match and cannot last during the upcoming intervals. Therefore, the phase error correction mechanism will fail to improve the timing accuracy in the absence of a stable rhythm. This consideration allows us to assume that in the presence of considerable interval discrepancies, the phase correction process is shut down or negligible, and the period correction is the only active mechanism. The correction of asynchrony comes to the play only after the subject has caught up with the tempo change.

It is thus reasonable that in this study dealing with a sudden step-change in tempo, we will only model the period error correction process.

## Experimental Approach

The current study uses the experimental approach from a previous study, where a more detailed version of the methodology and the experimental setup is explained (Darabi et al., 2010). Here a somewhat brief description is given, with specifications of modifications from the previous study. A finger-tapping task is given to a participant by presenting a click sequence over headphones. The participant is given the task of following the click sequence stimulus by tapping on the space bar of a MacBook Pro laptop computer. At some random point, the inter–click period is changed suddenly, and the response to these step changes is collected. In the previous study, 12 persons participated, and three different step changes were tested. Only three participants were used in the current experiment, but a more fine-grained range of 27 step changes were tested, each with 60–240 repetitions to reduce the effect of the noisy character of the data.

### Black-Box Approach

The internal workings of the synchronization mechanisms are still debatable due to the complex nature of the processes (Buhusi and Meck, 2005; Zatorre et al., 2007; Shadmehr et al., 2010). In the current work, we will not try to break down the system into smaller parts or speculate about the inner workings of the tempo perception and action processes. Instead, we will take a “black-box” approach of treating the system as a whole and assessing its behavior through a transfer function that describes the mathematical relationship between its input and output. The system would then be a human performing a rhythmic task. The input is the timestamps of onsets produced by the metronome before and after a tempo change, and the output is the sequence of taps generated by the participant in response to that change. The raw data must be processed before being used in our systems modeling approach as described in section “Data Preperation.”

### Experimental Setup

The sensorimotor task was finger-tapping in one-to-one to a regular sequence of heard impulses (via a headphone) and to keep the synchrony by coping with a new tempo as quickly as possible after its sudden introduction. The auditory sequences of input clicks of 3 ms long were generated by a computer application developed in Max/MSP [Computer software] (2018) and run under MAC OSX Lion, and generating the output was done by hitting the index finger on a MacBook Pro’s space button. The same computer was used for all experiments, and the time resolution for the registration was 1 ms. The total closed-loop delay was investigated by making the application respond to the impulse sounds (clicks) that were created by triggering an auditory impulse detector instead of pushing the space button. The round-trip delay was 10 ms, and it was estimated that also with the keyboard input device, the delay was less than 10 ms. As a result, the timestamps might have an error of maximum 10 ms.

To perform the task, three trained participants from the pool of 12 subjects in the previous study (Darabi et al., 2010) participated in the current experiment; two men (aged 31 and 33) and one woman (aged 32). The age of the participants was considerably below 66 and hence in the range that the errors of asynchrony are reported to be minimal (Drewing et al., 2006). Subjects were not particularly trained as musicians but were familiar with the test because of their participation in the previous study.

The test was carried out in a quiet room. To decrease the uncertainty of the motor action, the subjects were instructed to use the wrist and not arm and to take an abrupt, pulsed release of the downward force on the space key (Elliott et al., 2009). Both arrays of impulse timestamps generated by the application, and detected from user tapping, were recorded and saved in an XML format for post-processing.

**FIGURE 1:**
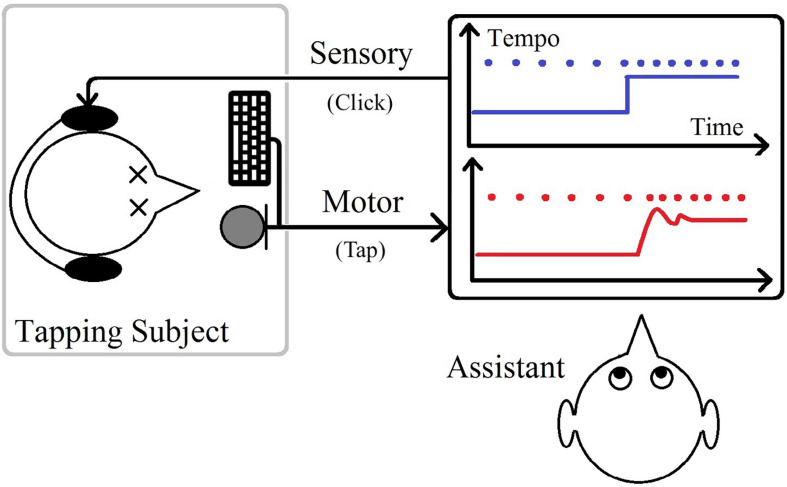
Illustration of the sensorimotor task.

The stimulus in this study emulated a step-metronome, a metronome jumping from one temporally regular (isochronous) sequence of click sounds to another. Each session included a randomized number of 27–40 repetitions between two tempos. Half of the repetitions, with a sudden decrease in tempo, were called positive steps due to an increase in the size of IOIs, and the other half, negative steps, had a sudden increase in the tempo. The number of clicks before a step-change was also randomized with the value changing between 10 and 30 throughout each session, to make the upcoming tempo change unpredictable for the participant. The participant would know that after a positive step, there would be a negative one with the same size, and vice versa, but could not know when the change would occur. Each participant took part in twenty-seven different tempo step sessions changing between 100 bps and a higher tempo (in a range of 102–200 bpm) back and forth. Each participant, for each of the tempo steps, took part in two to six sessions. Participants were blindfolded during all trials, and an assistant was present to monitor all experiments (Figure 1).

In the previous study the influence of some factors was found to be negligible, and as a consequence, those factors were kept constant in the current study. The effect of the lab conditions was studied in the previous work by carrying out the experiments in a normal quiet room as well as in an anechoic chamber. The effects of this factor were not statistically significant, and thus an anechoic chamber did not seem to be a necessity. Therefore, all trials in the current study were performed in a fairly damped and quiet room. Also, since other researchers reported that measurements using a keyboard might be subject to uncertainty in time recording (Shimizu, 2002), in the previous study, we examined two means of hand-clapping and finger-tapping in similar conditions and they led to indistinguishable results. Thus, only finger-tapping was chosen as the medium of motor act in this experiment.

Among four methods of up-sampling investigated in the analysis of the recorded trials in the previous study, a so-called cubic Hermite interpolation (PCHIP) was chosen, in which each piece between two samples is a third-degree polynomial^4^.

Narrowing down the experimental factors enabled us to cover a larger number of tempo changes and to collect many more repetitions from fewer participants in the current study. Michon showed in his step change study that by averaging the data over 10 tests, the noise of the inaccuracy was effectively reduced to a negligible component (Michon and Van der Valk, 1967). These numbers of repetitions were tried out in the previous study but were not considered sufficient to reduce the noise for the system identification methods used, especially for the small tempo steps. Small tempo steps required more repetitions due to the higher ratio of jitter and other noise compared to the size of the step. The number of repetitions collected for each step change, and each participant, were hence between 60 and 240 depending on the tempo change.

To summarize the comparison of data collection to the previous study, we chose a quiet but not anechoic room as environment; used finger-tapping on a computer keyboard for collecting the responses (clicks), and limited the experiment to three instead of twelve subjects. On the other hand, we expanded the number of tempo steps from 3 to 27 for each positive and negative jump and also collected hundreds instead of dozens of repetitions per each subject/tempo step to reduce the effect of random variations and thereby improve the input data to the model fitting.

## Data Preperation

The raw data collected from the experiment comprises of two sets of timestamps, of the stimuli “clicks” (which included a step-change in tempo) and the response “taps.” Since the experiment deals with a one-to-one clicking task, there should nominally be the same number of click and tap timestamps. Our black-box approach uses a SISO system model, and the properties/parameters of that system which best fit the observed data points are found with the same tool as in the previous study: an iterative function in MATLAB’s system identification toolbox called pem^5^ This function takes in two discrete-time signals as input and output (see section “Data Analysis”). As a first step of data preparation, the long sequences of clicks and taps in each session are split up into shorter sequences, one for each step-change in tempo. Then the timestamp data must be converted to time signals that can be used by this function.

### Breaking Down the Raw Data Into Repetitions

Each session was recorded as a long sequence of timestamps, collected for repetitions of the same step-change. The sequence was cut up into *N* sequences of single step repetitions with an increase in the IOI (known as positive steps) as well as *N* sequences with a decrease in the IOI (i.e., negative steps). The index *i* represented the repetition number. Each of the repetitions then contained a sequence of stimulus timestamps, *S*_*ij*_, for the *j*th click of the *i*th repetition, as well as a sequence of response timestamps, *R*_*ij*_ for the *j*th tap of the *i*th repetition.

The input sequences were cut so that they included two stimulus clicks at the pre-step tempo, but contained various numbers of clicks after the step. Since the corresponding response sequences were cut at the same points, they also had different numbers of post-step taps, but many enough that the responses were judged to come reasonably close to a stationary state after the introduction of the new tempo.

The raw data values, stimulus timestamps *S*_*ij*_, and the response timestamps *R*_*ij*_ were stored in two matrices, but given the different lengths of various repetitions, the values after the ending of shorter sequences were ignored.

The application produced the stimulus clicks as a metronome, so the stimuli *S*_*ij*_ was supposed to be the same as the nominal value, (=${\hat{S}}_{j}$) for all *i* and *j*. Although a ± 1 ms “noise” was detected due to the temporal resolution of the timestamping of the application.

**FIGURE 2:**
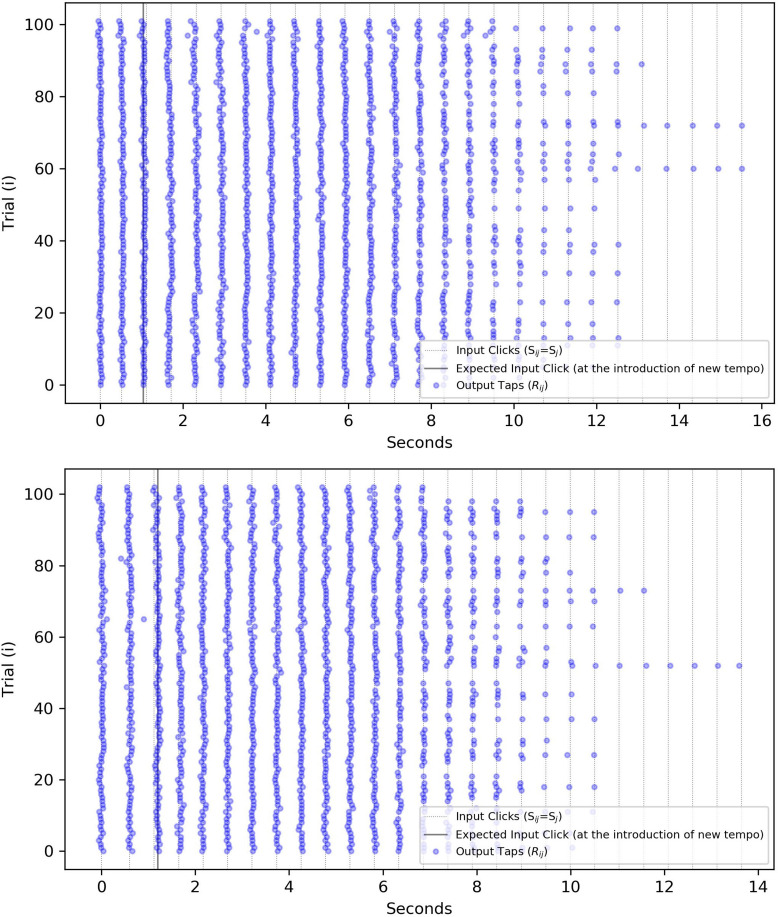
Visualization of the “event” variables for a specific subject/step size and for both positive/negative cases (Positive steps start in the range of 103–200 bpm and with an increase in ISI/IRI, or a decline in tempo jump to 100 bpm. Negative steps begin with 100 bpm in the tempo and with a decrease in the IOI, jump to a higher tempo.). (Top) A positive step: 102 repetitions of one participant’s responses to a tempo change from 115 to 100 bpm (increasing ISI from 0.522 to 0.6 s). The solid line shows where a stimulus onset was expected by the subject but occurred later due to the increase in ISI. (Bottom) A negative step: 103 repetitions of the same participant responding to the tempo change from 100 to 115 bpm (decreasing ISI). The solid line shows that the first click after the tempo change, arrived slightly later than expected. The horizontal axis is transformed to time instead of tap index to mark at which timestamp the input and output onsets have occurred.

Figure 2 visualizes the matrix *R*_*ij*_ by showing all repetitions for one subject and one specific tempo step against the nominal values of ${\hat{S}}_{j}$.

### Treating Multiple Taps and Missed Taps

Sometimes a participant produced more than one tap for one particular stimulus click. We had to make sure that the index *j* of the output *R*_*ij*_ was associated with the correct input *S*_*ij*_. Therefore, for each repetition *i* we let only the tap closest to the nominal ${\hat{S}}_{j}$ be indexed as *R*_*ij*_, which automatically excluded accidental double-tap responses.

It also happened that a participant performed the tapping too softly, such that the impulse detecting sensor would not register the created response (a space key in this case). By the rule described above, a missing tap would cause the tap before or after itself to be assigned to two consecutive indices, *R*_*ij*_ and *R*_*ij*__+__1_. An additional rule was then proposed that each tap should have only one index, and in the case of *R*_*ij*_ = *R*_*ij* + 1_, the closest nominal stimulus to the tap’s timestamp, between ${\hat{S}}_{j}$ and ${\hat{S}}_{j+1}$, would define its index, while the other one will be assigned a *NaN* value^6^.

### Aggregating Response Data Over Repetitions

To convert matrices *S*_*ij*_ and *R*_*ij*_ to time signals that can be used by the model fitting algorithm, they should first be aggregated over the repetitions *i* into vectors ${\hat{S}}_{j}$ and ${\hat{R}}_{j}$. This is trivial for the input stimuli because the metronome timestamps vary negligibly (±1 ms) over repetitions of the same step size, i.e., ${\hat{S}}_{j}≃S_{i\,j}$ for any *i*. Aggregating the matrix *R*_*ij*_ into a representative ${\hat{R}}_{j}$ array for all repetitions was done as follows.

First, after the re-indexing process described in section “Treating Multiple Taps and Missed Taps,” all response events, *R*_*ij*_, events that were assigned to the same index *j* were grouped in the cluster. *R*_*j*_ = {*R*_*ij*_|∀*i*}

Next, outliers were removed within each cluster *R*_*j*_. Sometimes, a tap was mistakenly performed off-phase due to a lack of attention or some other reason. Such taps that were three standard deviations away or more from the mean of *R*_*j*_, were marked as outliers and excluded. This led to the exclusion of around 1.7% of the responses/taps in our dataset^7^.

After grouping events and excluding outliers, a single value should represent all taps in each cluster, *R*_*j*_, across the repetitions. With the further considerations, the mean across repetitions *i* for each *R*_*j*_, was considered an acceptable candidate. We know from the properties of asynchrony in rhythmic tasks that the distribution of the responses/taps is not always Gaussian around an intended timestamp. Instead, the deviation from the intended timestamp follows a power law with an exponent that depends on the rhythm frequency (Hänggi and Jung, 1995). Such a “colored noise of asynchrony” is known as a 1/*f*^*B*^ characteristic (Torre and Wagenmakers, 2009). Because our trials were between 100 and 200 bpm, the maximum tempo was only twice the minimum, and for such a narrow range of inputs, the color of the noise will not have a significant influence on the calculation of the mean value. Therefore, we ignored such spectral aspects of the noise. Furthermore, such 1/*f*^*B*^-noise is attributed to sustained attention processes and fatigue in long trial sequences (Pressing and Jolley-Rogers, 1997), but trials in our experiment were divided by random breaks to reduce the effect of fatigue on the performance. Distribution of asynchrony in other isochronous finger-tapping experiments, which are performed on a similar limited range of tempos, has also been shown to be Gaussian (Aschersleben, 2002). Based on these considerations, we assumed that we could represent the set of all taps that correspond to the same tap index by their arithmetic mean, ${\hat{R}}_{j}$.

Note that in the same way that we can aggregate one participant’s data over all repetitions of the same step size and direction, we can also aggregate over the pool of data from the three participants, although still for the same step size and direction.

### Calculating ISI and IRI Based on Aggregated Events

As discussed in section “Event and Interval Variables and the Choice Between the Period Error Correction and Phase Correction Process” concerning the nature of the SMS task at hand, a sudden change in the interval, the focus would be on the period error correction and not the phase error correction process. This preference implies that instead of focusing on the properties of asynchrony between the input stimulus and output response, ${\hat{S}}_{j}$ and ${\hat{R}}_{j}$, as aggregated event variables, we are interested in the comparison between the values of interval variables.

We can obtain the aggregated intervals by calculating the time difference between two consecutive values of the aggregated events, ${\hat{r}}_{j}={\hat{R}}_{j}-{\hat{R}}_{j-1}$, and ${\hat{s}}_{j}={\hat{S}}_{j}-{\hat{S}}_{j-1}$. In the same way as for the event variables, interval values that deviated more than three standard deviations from each mean were considered as outliers and were discarded. This leads to the exclusion of another 2.4% of datapoints in our dataset.

### Transforming Index Number to Time

The aggregated ISIs and IRIs have so far been denoted with index number *j*. The next step is to transform the index number to the actual time of the events. This transformation is demonstrated for one example of data points, as given in Table 1, showing that ISIs (${\hat{s}}_{j}={\hat{S}}_{j}-{\hat{S}}_{j-1}$) can be described as a function of the time of ${\hat{S}}_{j}$ instead of the index *j*. Similarly, IRIs (${\hat{r}}_{j}={\hat{R}}_{j}-{\hat{R}}_{j-1}$) are expressed as function of the time ${\hat{R}}_{j}$^8^.

**TABLE 1:** One example of data points: the event times, ${\hat{S}}_{j}$ and ${\hat{R}}_{j}$, are for 5–6 clicks/taps during an IOI step of 0.522–0.6 s (a tempo change from 115 to 100 bpm).

j	${\hat{S}}_{j}$	${\hat{R}}_{j}$	${\hat{s}}_{j}$	${\hat{r}}_{j}$
0	0	0.011	–	–
1	0.521	0.533	0.521	0.522
2	1.12	1.053	0.599	0.521
3	1.719	1.687	0.599	0.624
4	2.32	2.311	0.601	0.633
5	2.919	2.923	0.599	0.611

*Due to the small temporal uncertainty of the experimental system the clicks, ${\hat{S}}_{j}$, do not occur exactly at an integer multiple of 0.522 s or 0.600 s. The interval variables, ${\hat{s}}_{j}$ and ${\hat{r}}_{j}$ are computed as the difference between consecutive events.*

### Upsampling the Signals

**FIGURE 3:**
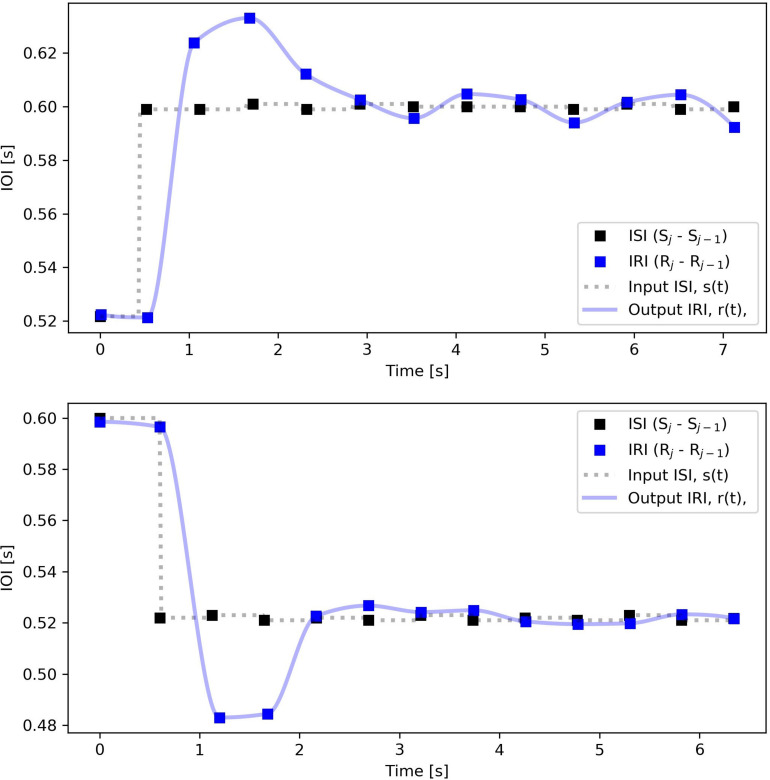
Aggregated stimulus and response intervals over repetitions for the same subject/step size as Figure 2 and Table 1. ISI, ${\hat{s}}_{j}={\hat{S}}_{j}-{\hat{S}}_{j-1}$ (■), and IRI, ${\hat{r}}_{j}={\hat{R}}_{j}-{\hat{R}}_{j-1}$ (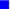), are plotted as function of time instead of index number. The output *r*(*t*) is upsampled with the PCHIP algorithm and the input *s*(*t*) with staircase interpolation, both at 60 Hz sampling rate. Note that for the positive steps (increasing IOI, decreasing tempo) the arrival of the oversampled input step is marked slightly (This shift is only considered for positive steps where IOI increases. It is the same as the temporal distance between the solid line and the third dashed line in Figure 2, which in seconds amounts to 60/endtempo(bpm)−60/starttempo(bpm)) earlier than the arrival of the first onset of the new rhythm.

The representation of the average IRI, ${\hat{R}}_{j}$, is smoother than any of the individual repetitions, but is still a sequence of discrete events. At this stage, we upsample the input ISI and output IRI data to signals with a considerably higher time resolution considerably higher than IRI or ISI intervals, by regularly inserting several values in between these irregularly sampled data-points. This conversion assumes a short enough “time quantum” as the indivisible unit of time that can be shared among all trials and will result in time signals with a regular, but higher frequency of sampling, namely *s*(*t*) and *r*(*t*), respectively, for stimulus and response, as seen in Figure 3. These new signals, although still discrete-time signals, are quite smooth and represent the hypothetical continuity of the internal timekeeper’s tempo with a higher regular frequency of sampling. In the previous studies (Darabi et al., 2010), given the advice of sampling at ten times the dominant frequency of the system (Söderström and Stoica, 1989; Ljung, 1999) we chose the up-sampling frequency as 60 Hz. Also, four different methods of interpolation were computed: staircase, linear, cubic spline, and shape-preserving piecewise cubic. Among these four, shape-preserving, or PCHIP, was chosen to sample the output IRI in which the piece between two samples is interpolated with a third-degree polynomial. In this work, we use the same interpolation method for IRI outputs. For the input ISIs, we use a staircase interpolation with some considerations as explained in section “Defining the Time of the Step-Change” on where to mark the timestamp of the step change.

### Defining the Time of the Step-Change

During each click sequence, the participant will develop an anticipation of when the next stimulus click is supposed to come. Therefore, a change in tempo will be detected differently in the two cases: either a new click arrives earlier than anticipated, which happens in the case of a step-down in IOI (a step-up in tempo), or a new click does not come at the expected time, which occurs during a step-up in IOI (a step-down in tempo).

Due to this consideration, we define the start of a step differently for negative and positive cases. For the negative steps, where a click arrives earlier than anticipated, the timestamp of that click’s arrival will define the start of the step-change^9^ For the positive steps, however, we define the start of the step-change to be when the click was anticipated but did not arrive, instead of the late arrival of the click, e.g., the dashed line in Figure 2 (bottom) instead of the third solid line where the first onset of the new rhythm has arrived. This replacement is also visible in the negative step of Figure 3 and will impact the quantity of the delay reported in the model fitting (see “Results” section).

Also, the model fitting algorithm assumes that the response to a step input always happens sometime after the step has occurred. Therefore, in order to make sure that the jump in the oversampled IRI signal certainly happens after the step-change definition, we offset the IRI signal by adding a large enough processing delay (1 s, for example). This value will later be subtracted from all the delays estimated by the model fitting algorithm.

### Normalization

**FIGURE 4:**
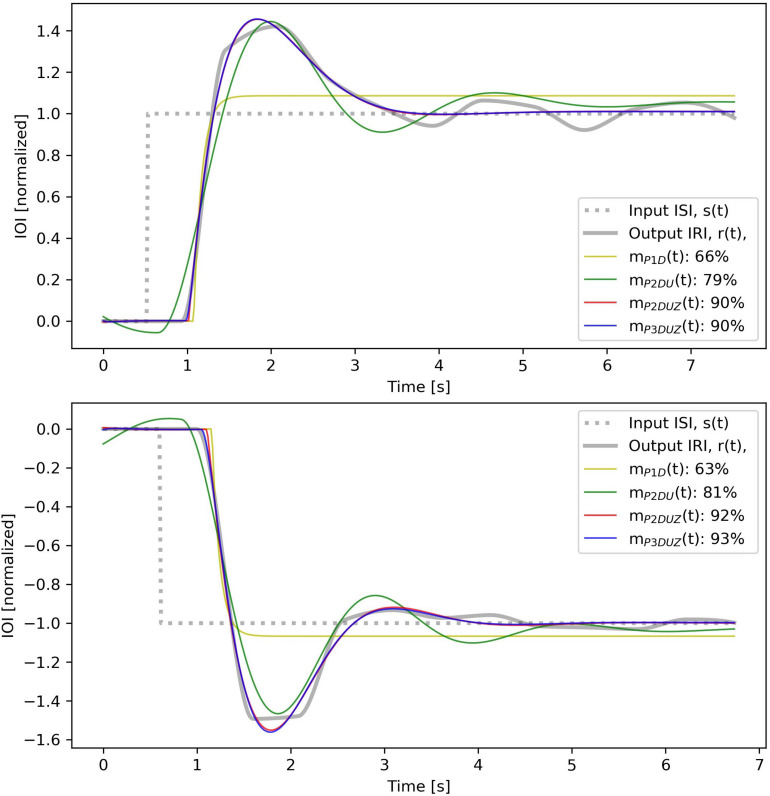
Measured data, ISI and IRI, as well as output signals from simulations with the four models defined in section “Data Analysis.” The input *s*(*t*) and the output *r*(*t*) amplitudes are normalized. In addition, a processing delay of 0.4 s has been added to the output signal, before the model fitting.

The model fitting algorithm assumes that all signals, input and output, have the value zero before the time zero. In order to satisfy this condition, we scale signals *s*(*t*) and *r*(*t*), with the same scale factor, so that the input signal *s*(*t*) begins with 0 and ends with +1 for positive or −1 for negative steps, as seen in Figure 4.

At this stage, the upsampled ISI and IRI signals are ready to be provided to the model fitting algorithm, which is the pem function in MATLAB’s System Identification Toolbox^TM^, as mentioned earlier. This function, by minimizing the normalized root mean square error (NRMSE), estimates parameters that produce modeled output curves to fit as close as possible to the observed signals.

## Data Analysis

The stimulus input, *s*(*t*), and the response output, *r*(*t*), prepared as described in the previous section, are functions of time, but a Laplace transformation allows a formulation of the system model in the complex frequency domain (the s-domain), as described in section “Approach in the Present Study.” Such a model describes the transfer characteristics from the input to the output signals (Widder, 2015). The theoretical model that describes the relationship between these two is written in the complex frequency domain as a function of *s* = σ + *j*ω, after applying a Laplace transformation and in the form of a rational transfer function, *G*(*s*) = *A*(*s*)/*B*(*s*), where *A*(*s*) and *B*(*s*) are polynomials (Trumper, 2004). The zeros of the system are the roots of the numerator polynomial, *A*(*s*), and the poles are the roots of the denominator, *B*(*s*).

**FIGURE 5:**
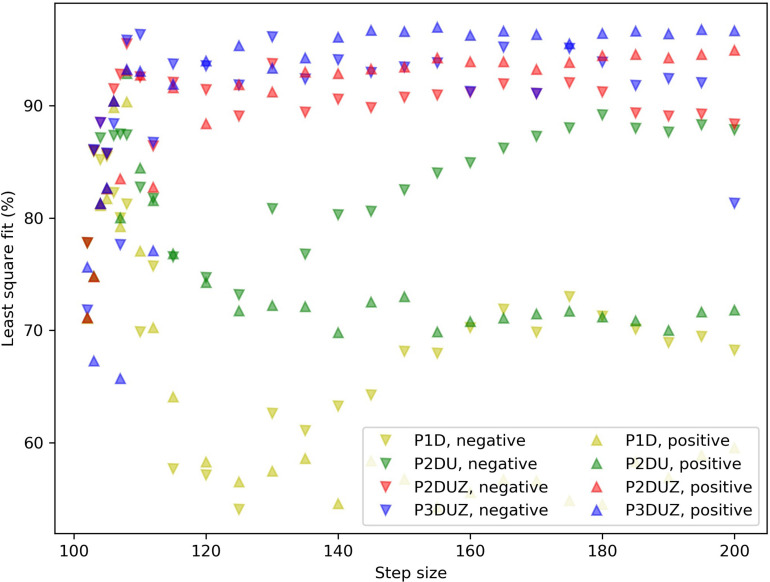
Fit ratios for four models (P1D, P2DU, P2DUZ, and P3DUZ), on data aggregated over three participants. Negative steps are depicted with (∨) and positive ones with (∧).

As mentioned in section “Approach in the Present Study,” the used software tool for fitting model parameters to experimental data allows a model complexity up to three poles and one zero. We will start with the simplest form of transfer function with a gain, one real pole, and a delay. Then we will add more poles and a zero to improve the fit. This step-by-step increase in the complexity reveals how each added parameter can capture a qualitative feature in the observed signals, and thereby improve the least-squares fit^10^ Fit ratios, normalized root mean squared errors, for four different models are given in Figure 4, for one example, which is the same case as in Figures 2, 3. Figure 5 depicts how much the inclusion of each parameter would improve the fit ratio across all step sizes, where each step contains data merged from three participants. It is unsurprising that including more model parameters improves the fit; however, not all parameters play the same role. We will introduce them in this section and discuss them in the results section separately to demonstrate how different parameters play different roles in their qualitative impact on the shape of the signal by adding features such as delay, oscillation, overshoot without a following undershoot, etc.

### The First Real Pole and a Time Delay (P1D)

We begin with a rational transfer function, *G*(*s*), that has a proportional gain *K*_*p*_ and a time constant *T*_*p*__1_. Typically, the output follows the input after some delay, so we also define a “transport delay” *T*_*D*_ in the time domain which is represented by an *e*^−*T*_*D*_*s*^ term in the complex frequency domain^11^.

(1) $$G_{P\,1\,D}\,\left(s\right)=K_{p}\,\frac{1}{1+T_{p_{1}}\,s}\,e^{-T_{D}\,s}$$

(2) $$p_{1}=\frac{-1}{T_{p\,1}}$$

This system, which allows a single pole *p*_*1*_ and a transport delay *T*_*D*_, is called P1D. As shown in Figure 4, this model cannot generate an oscillation, which leads to a low fit ratio of 66 and 63% for the positive and the negative examples.

### Adding the Second Pole (P2DU)

In the example of Figure 4, the output *r*(*t*) for the P2DU model shows an overshoot and a subsequent oscillation around the input *s*(*t*), whereas the P1D can not capture this quality. Exhibiting an overshoot is a sign of an *oscillatory system*, which needs at least two complex poles, i.e., (1 + *T*_*p*_1_*s*_)(1 + *T*_*p*_2_*s*_) in the denominator, where *p*_1,2_ = −1/*T*_*p*_1,2__. This denominator term can be rewritten in the form of a second-order polynomial, based on a period *T*_ω_ (alternatively described by the frequency of oscillation *f*_ω_) and a damping ratio ζ:

(3) $$G_{P\,2\,D\,U}\,\left(s\right)=K_{p}\,\frac{1}{1+2\,\zeta \,T_{\omega }\,s+{\left(T_{\omega }\,s\right)}^{2}}\,e^{-T_{D}\,s}$$

(4) $$p_{1,2}=\frac{-\zeta \pm \sqrt{\zeta^{2}-1}}{T_{\omega }}$$

(5) $$T_{\omega }=\frac{1}{f_{\omega }}=\frac{2\,\pi }{\omega }$$

If ζ < 1, then according to Eq. 4, poles will have imaginary values and the system, known as underdamped^12^ will oscillate and exhibit an overshoot. In overdamped systems (ζ > 1), both poles are real, and the output will follow the input without oscillation.

### The Zero (P2DUZ)

If α < 1, a second-order oscillator such as P2DU, in addition to an overshoot of the size α, also shows a subsequent undershoot of the size of α^2^, followed by an overshoot of α^3^, etc. This is because this model only has a frequency of oscillation and a damping ratio, which causes the system to show a secondary undershoot of the same peak ratio as that of the initial overshoot.

In order to capture the more complex response (overshoot without a following undershoot), we introduce a zero, an extra term in the numerator of the transfer function (Trumper, 2004):

(6) $$G_{P\,2\,D\,U\,Z}\,\left(s\right)=K_{p}\,\frac{1+T_{z}\,s}{1+2\,\zeta \,T_{\omega }\,s+{\left(T_{\omega }\,s\right)}^{2}}\,e^{-T_{D}\,s}$$

(7) $$z=-\frac{1}{T_{z}}$$

According to Eq. 7, any of the previous systems without a zero, have *T*_*z*_ = 0 and can be thought as having an infinite *z*. As seen in both Figures 4, 5, introducing a zero can lead to a substantial improvement in the fit ratio.

### The Third Pole (P3DUZ)

Finally, a third pole can be given by a third-order denominator; multiplying the complex conjugate pair with another linear term to achieve the most general form of the model:

(8) $$G_{P\,3\,D\,U\,Z}\,\left(s\right)=K_{p}\,\frac{1+T_{z}\,s}{\left(1+2\,\zeta \,T_{\omega }\,s+{\left(T_{\omega }\,s\right)}^{2}\right)\,\left(1+T_{p\,3}\,s\right)}\,e^{-T_{D}\,s}$$

(9) $$p_{3}=-\frac{1}{T_{p_{3}}}$$

According to Eq. 8 depending on the complexity of the model we have up to six parameters to study in the “Results” section: the gain *K*_*p*_, the delay *T*_*D*_, the first two poles represented by *T*_ω_ and ζ, the third pole defined by *T*_*p*__3_, and the zero defined by *T*_*z*_. Table 2 shows the inclusion of these parameters in each model.

**TABLE 2:** Model parameters to describe the relationship between ISI and IRI.

Model	P1D	P2DU	P2DUZ	P3DUZ
Gain	*Kp*	*Kp*	*Kp*	*Kp*
*P*_1,2_	*T*_*p*__1_	*T*_ω_	*T*_ω_	*T*_ω_
*P*_1,2_	–	ζ	ζ	ζ
Delay	*T*_*d*_	*T*_*d*_	*T*_*d*_	*T*_*d*_
*Z*	–	–	*T*_*z*_	*T*_*z*_
*P*_3_	–	–	–	*T*_*p3*_

## Results

In this section, in order to describe the relationship between input ISI and output IRIs, we will estimate the model parameters of the transfer function in the Eq. 8, i.e., gain

*K*_*p*_, delay *T*_*D*_, up to three poles defined by *T*_*p*__1,2,__3_, and one zero *T*_*z*_. Their qualitative impacts on the shape of the response signal, as well as their trends, as a function of step size will also be discussed.

### General Characteristics of Step Response Signals in Time Domain

Figure 5 depicts the normalized least square fit for 27 negative and positive steps, aggregating over the three participants. We can see that moving toward more complex models improves the fit overall.

**FIGURE 6:**
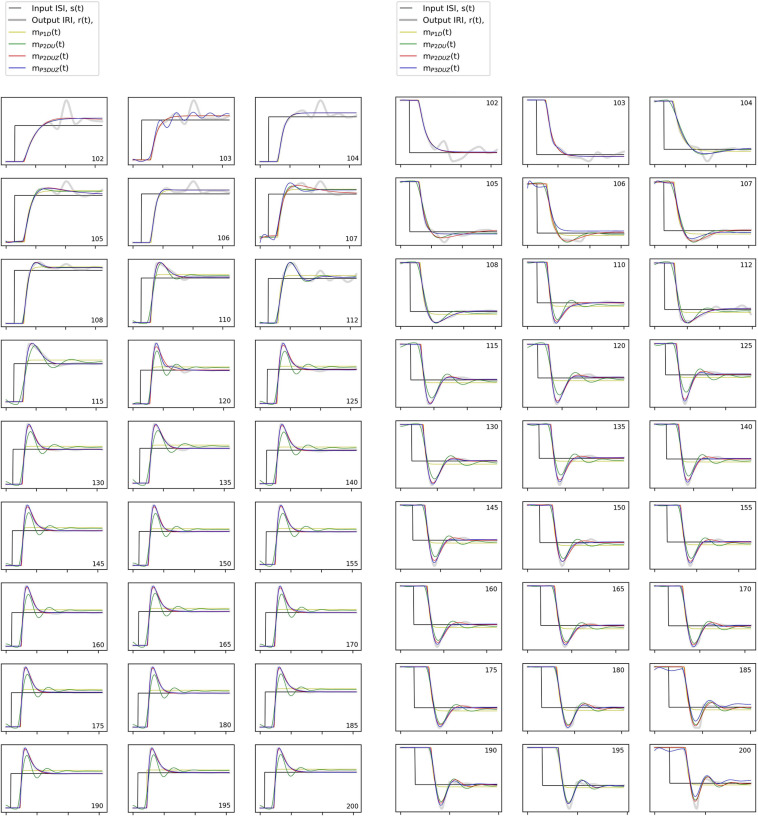
The stimulus signal, *s*(*t*) (black), observed step response aggregated over participants, *r*(*t*) (thick gray signal), and modeled response for all models, *m*_*P*__2_*_*DU*_*(*t*), *m*_*P*__2_*_*DUZ*_*(*t*), and *m*_*P*__3_*_*DUZ*_*(*t*) (thin colored curves). The axes are the same as in Figure 4 with a normalized unit step input. The numbers in the corner of the charts represent the initial tempo for the positive steps (left) and the destination tempo for the negative steps (right) in bpm, while the base tempo is 100 bpm.

In Figure 6, corresponding time signals are shown for all tempo steps aggregated over three participants, both negative (left) and positive (right). The black curve, *s*(*t*), shows the stimulus step input, the thick gray curve depicts the observed aggregated response, *r*(*t*), and thin curves with the same color code as Figures 4, 5 show how each model produces the step response. We can observe that *m*_*P*__2_*_*DU*_*(*t*), *m*_*P*__2_*_*DUZ*_*(*t*), and *m*_*P*__3_*_*DUZ*_*(*t*) can capture the overshoot due to the inclusion of two complex poles (P2) and the letter U. All models include the letter D and thus capture the delay. The models P2DUZ and P3DUZ, due to the inclusion of a zero (Z), allow for a proportionally smaller undershoot after the initial overshoot.

As a general observation, the model parameters will vary across step sizes. Such dependence indicates a non-linear system, which is a common observation in SMS research (Schulze et al., 2005; Bavassi et al., 2013). This non-linearity means that the same LTI system, no matter its complexity, cannot account for modeling a single participant’s SMS behavior in response to a sudden step-change in IOI. However, toward the end of this section, we will introduce one model that allows changing its parameters with the change in step size, as a workaround for the non-linearity problem. We will limit that model to the step-changes of 12% or higher. This limitation is because despite collecting more repetitions for smaller steps (in the range of 102 to 110 bpm^13^, the response signals and their modeled curves in Figure 6 still appear noisier than for the larger steps, as it can also be seen with the lower fit ratios in Figure 5.

**FIGURE 7:**
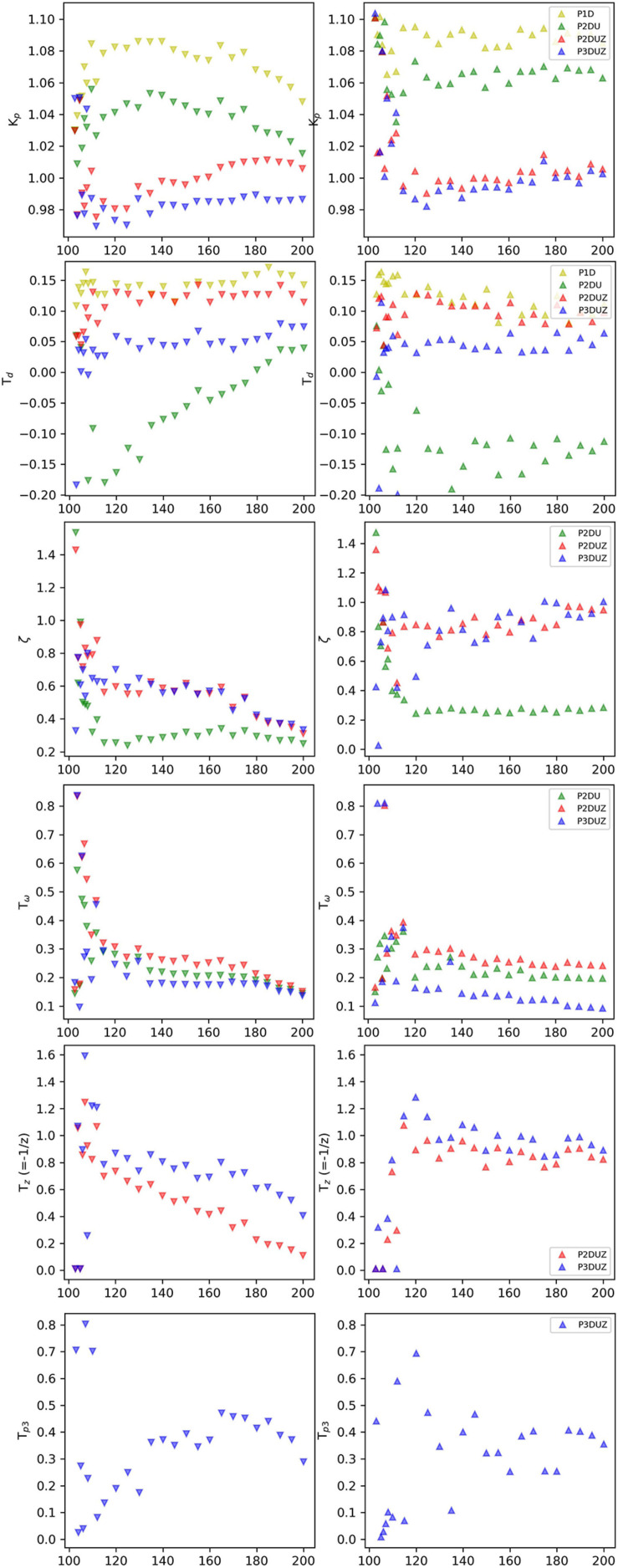
Gain (*K*_*p*_), delay (*T*_*D*_), damping ratio (ζ), oscillation period (*T*_ω_), zero (*T*_*Z*_-values), and the third pole (*T*_*P*__3_-values). The parameters of the aggregated data over participants for negative (∨) and positive (∧) steps, as reported by the relevant models.

Figure 7 reflects a regime shift at around 112 bpm, especially for the models P2DUZ and P3DUZ. The transition at this tempo, which matches the threshold between the subliminal and supraliminal steps, demonstrates that our “black-box approach” that disregards the system’s inner workings, can still shed light on how different brain structures may be involved in an SMS task, and is in agreement with neurophysiological insights regarding different correlates involved in adaptation to subliminal or supraliminal changes.

Some previous behavioral studies indicate that period and phase correction processes are separate behaviors, with the former being a higher-level cognitive process, while the latter an automatic and related to lower-level cognitive processes (Repp and Su, 2013). Repp (2002); Repp and Penel (2002) showed that period correction is under conscious control, while phase correction can be diminished by conscious effort although never shut off completely. Friberg and Sundberg (1995) claimed that the occurrence of overshoot in response to a step-change in tempo does not depend on the amplitude of the step-change, but rather on the awareness that the step-change has taken place. Schulze defined two linear models which used a different terminology by calling the phase and period correction respectively “asynchrony-based” and “interval-based” and showed that even if an asynchrony-based model matches the data qualitatively, to fit the data quantitatively, we need to consider another, interval-based correction process that adjusts the period, although, it can switch on and off during a trial (Schulze et al., 2005).

There are also neuroscientific studies pointing to different neural correlates of subliminal vs. supraliminal errors. Bijsterbosch et al. (2011a, b) focused on the role of the *cerebellum*, the part of the brain at the back of the skull controlling muscular activity. Their fMRI imaging showed that the right cerebellar dentate nucleus and primary motor and sensory cortices were activated during regular timing and during the correction of subliminal phase errors. The correction of supraliminal phase errors led to additional activations in the left cerebellum and right inferior parietal and frontal areas. They also showed that suppression of the left but not the right cerebellum with theta burst TMS^14^ significantly affected supraliminal error correction (Bijsterbosch et al., 2011b). More recent work provides clues that different cortical mechanisms may be employed when correcting for errors in SMS that increase tap-tone asynchrony compared with those that decrease it (Jantzen et al., 2018). Although the specific involvement of the left cerebellum in the correction of supraliminal errors is widely agreed upon, other neural structures such as prefrontal and frontal motor areas and the *basal ganglia* (Zatorre et al., 2007) are reported to show differential involvement in subliminal vs. supraliminal error correction (Praamstra et al., 2003; Thaut M. et al., 1998; Thaut et al., 2009; Schwartze et al., 2011).

### The Gain Factor (*K*_*p*_)

The experiment is based on a 1:1 synchronization task, and the stationary response signal settles and approaches the input signal. Therefore, the gain *K*_*p*_ takes a value around 1 as expected^15^, see Figure 7, the first row, for negative and positive steps.

### The Delay Parameter (*T*_*D*_)

It takes time to perceive and react to a sudden change in the tempo, so the model fitting algorithm is expected to report a positive delay. After the considerations in section “Defining the Time of the Step-Change,” illustrated by Figure 3, the delay is measured with respect to when the step jump in the input signal is defined, i.e., the first chance the participant has to detect a change and differs for the two step directions. A processing delay was added, as described in section “Defining the Time of the Step-Change,” so the same delay has to be subtracted from the model fitting algorithm’s estimate of *T*_*D*_. The results in Figure 7 (the 2nd row) show that despite this up/down-difference, the three models, P2DU, P2DUZ, and P3DUZ, lead to similar values for negative and positive steps.

P2DU is constrained to assume the following oscillations after the initial overshoot, affecting the estimation of the system’s delay. As a result, after subtracting the processing delay, this model exceptionally reports negative values for the delay. For the three other models, the model fitting estimates a relatively constant delay for the supraliminal tempo steps equal to or above 112 bpm, where the size of the steps, exceeds around 12% for negative steps and 11% for positive ones. For the subliminal range, i.e., below the threshold for conscious perception (Thaut M. et al., 1998; Repp and Steinman, 2010), the estimates are too noisy to justify a constant value for the entire range. Table 3 gives the mean value of delays that resulted for P2U, P2DUZ, and P3DUZ, for the range equal to, or above, 112 bpm.

**TABLE 3:** Mean delay values for supraliminal step changes (±115 bpm or larger) as estimated by three models, extracted from aggregated data over three participants.

Model	*Td* Negative	*Td* Positive
P1D	145 ms	111 ms
P2DU	125 ms	101 ms
P2DUZ	54 ms	45 ms

### Damping Ratio (ζ)

Figure 7 (the 3rd row) shows the damping ratio of aggregated data over three participants for negative (∨) and positive (∧) steps, as estimated for the P2DU, P2DUZ, and P3DUZ models. Note that according to Eqs 3, 6, and 8, this parameter is defined only for models containing at least two poles and is absent from Eq. 1 associated with P1D.

We can observe that there is some disagreement in how different models estimate the value of the damping ratio as it can be affected by the introduction of a zero, or a third pole. However, while within the noisier subliminal range, it can sometimes exceed one, all models estimate it below 1 for tempo steps above 112 bpm. As described in section “The Zero (P2DUZ),” ζ = 1 is the critical boundary between the underdamped and overdamped systems. From a dynamic system point of view, when ζ < 1, the underdamped system exhibits the oscillation of the output around the step input, which causes a more visible overshoot in the step response, as seen in the time signals for all step changes above 112 bpm in Figure 6.

Such a qualitative distinction between the two subliminal and supraliminal regimes has been made in research on SMS. Repp (2003) and Thaut M. et al. (1998) have reported an overshoot in a human’s response to supraliminal tempo step-changes above consciousness of sensation. Such an initial overshoot is reportedly caused by an overestimation of the new tempo (Repp, 2005; Repp and Su, 2013), before synchronization occurs within a few taps (Mates, 1994). However, for the subliminal steps, only a gradual adaption to the new rhythm was observed, without any detectable overshoot, Repp (2010) and Thaut and Kenyon (2003). Friberg and Sundberg (1995) have also discussed the presence or the absence of an overshoot based on the awareness of the step-change having taken place or not. They argued that for a period correction process to take place, there has to be an awareness that the step-change has occurred. They have concluded that period error correction can be a higher-level cognitive process, while phase correction is automatic and related to lower-level cognitive processes. The differentiation of the responses between supraliminal and subliminal tempo could be associated with different brain circuitry, further discussed in the discussion part (section “Future Work”).

Our results also show that the overcorrection in the supraliminal range consistently occurs even when the tempo doubles or halves, from 100 to 200 bpm and vice versa. This observation is consistent with Repp (2011) who reported overcorrections of IRIs for the cases where the IOIs changed as much as a tripling from 400 to 1200 ms.

### Oscillation Period (*T*_ω_)

The next parameter is the oscillation period, *T*_ω_, which, similar to the damping ratio, is only valid for models with at least two poles. Despite the expected noisier results within the subliminal range, Figure 7 (the 4th row) shows that the oscillation period for aggregated data can sometimes exceed a certain threshold within this range. A larger oscillation period, or, equivalently, a lower frequency of oscillation (*f*_ω_), means a longer time of adaptation to the tempo, i.e., a larger number of taps after the introduction of tempo step.

The behavioral difference between the subliminal and supraliminal ranges is, in this case, also in agreement with the literature. Thaut M. et al. (1998) have reported that adaptation to step-changes exhibited different patterns in these two regimes, adapting rapidly after large step-changes but only very gradually after small step-changes. Hary and Moore (1985) also showed with computer simulations that for subliminal step-changes, period correction gets very slow and gradual. Thaut and Kenyon (2003) reported similar results in an antiphase tapping step-change experiment. In the synchronization of rhythmic arm movements to a syncopated metronome, small tempo shifts of +2% or −2% were inserted in the metronome’s stimulus frequency, and the response interval showed a more rapid adaptation to the frequency-incremented stimulus period (Repp, 2005).

### Zero (−1/*T*_*z*_) and Overshoot Without Undershoot

Another quality of the output signal *r*(*t*), as seen in Figures 4, 6, is that even if there is a considerable overshoot, the output will settle with a minor or non-existing undershoot [in agreement with Pressing (1998, 1999)]. This is true for both negative and positive steps.

As mentioned in section “The Zero (P2DUZ),” the inclusion of two complex poles can allow for an overshoot but cannot kill off or reduce the size of a second undershoot when the modeled output oscillates around the input step. This quality is observable in Figure 4, where the P2DU modeled output (the green curve), as opposed to the actual input, assumes a second and even a third undershoot until the model adapts to the step-change^16^ The blue and red curves (P2DUZ and P3DUZ) in Figure 4, however, display an insignificant, or a relatively smaller, undershoot in accordance with the observed signal. The ability of the model to exhibit an overshoot without an undershoot of the same ratio implies the existence of an additional zero^17^ see section “The Zero (P2DUZ).”

Figure 7 (the 5th row) shows the estimated zero for the aggregated data over three participants. Values of *T*_*z*_ are positive, meaning that the zero ($z=-1/T_{z}=x+i\,y$) is in the left half of the complex frequency plane. As the zero’s absolute value increases, and it moves further into the left half-plane (LHP), the step response of this system starts to resemble the step response of the system without a zero. The time-domain effect of a LHP zero on the step response is to increase the overshoot, decrease the peak time, and decrease the rise time; the settling time is not affected too much. In other words, a LHP zero makes the step response faster (Baryshnikov, 2014).

### The Third Pole (−1/*T*_*p*__3_)

The last parameter is only valid for the P3DUZ model. This additional parameter will increase the fit ratio compared to simpler models, including P2DUZ, as seen in Figure 5. The last row of Figure 7 shows the value of the third pole as a function of the step size for data aggregated over the three participants.

### A Unified Model

We have so far observed how each of the model parameters vary systematically with the step-change size. In an attempt to present a unified model that accounts for all step-changes, we can derive linear relationships for each parameter’s dependency on the step size. We represent the independent variable, or the step-change size, in its relative form. Therefore, we obtain Δ, or the percentage of relative change in tempo from *t*_*start*_ to *t*_*end*_, from the Eq. 10, for negative and positive steps:

(10) $$\Delta_{\mp }=\frac{\pm t_{e\,n\,d}\mp t_{s\,t\,a\,r\,t}}{t_{s\,t\,a\,r\,t}}$$

**FIGURE 8:**
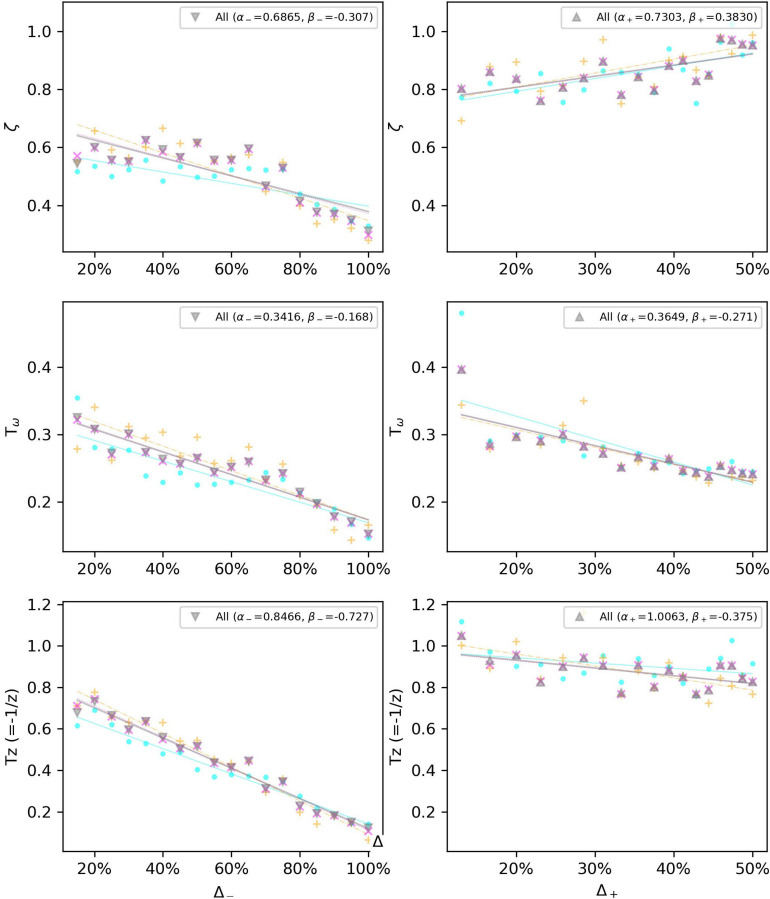
Linear regression estimates for the observed values of the P2DUZ model parameters, i.e., delay (*T*_*D*_), damping ratio (ζ), oscillation period (*T*_ω_), and zero (–1/*T*_*Z*_), for negative and positive step sizes in the supraliminal range (base tempo = 100 bpm).

**FIGURE 9:**
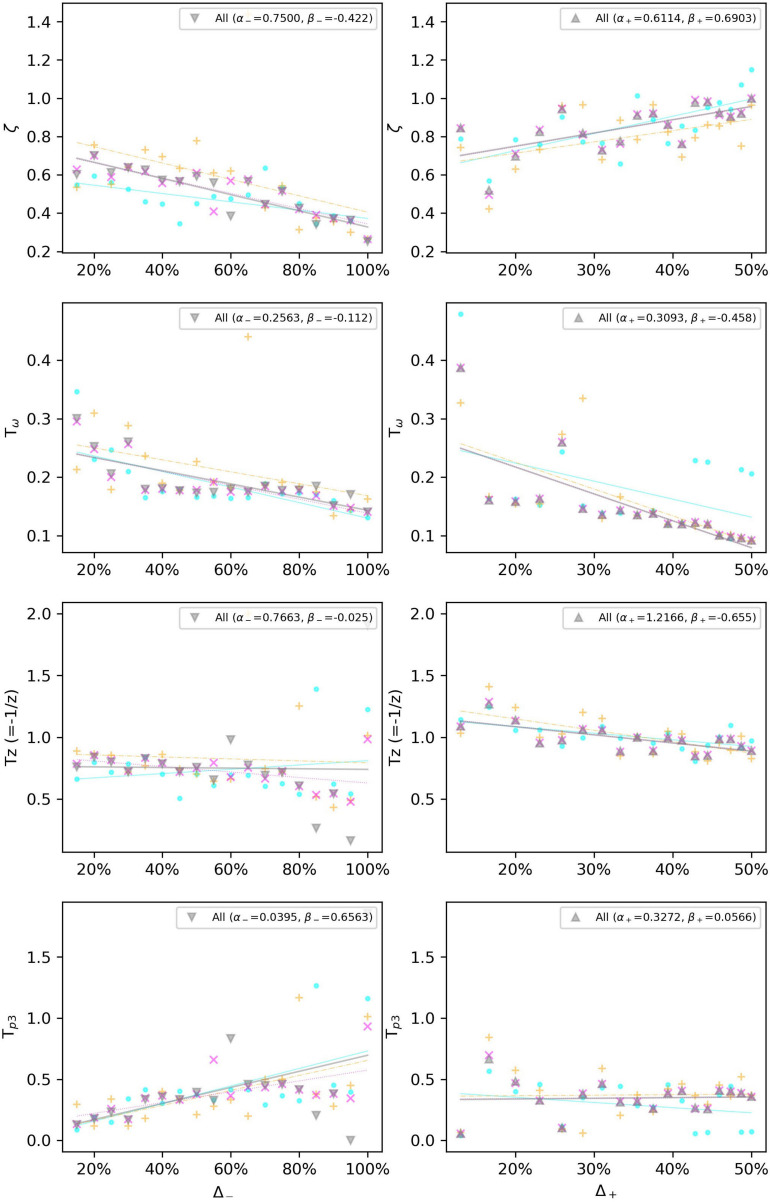
Linear regression estimates the observed values of the P3DUZ model parameters, i.e., delay (*T*_*D*_), damping ratio (ζ), oscillation period (*T*_ω_), zero (–1/*T*_*Z*_), and the third pole (–1/*T*_*p*__3_), for negative (up to 100% increase in tempo) and positive step sizes (up to 50% decrease in tempo) in the supraliminal range (base tempo = 100 bpm).

Due to the noisy measurement for subliminal step-changes, we will limit our model only to the supraliminal range, equal to or above step changes related to 115 bpm, i.e., Δ_–_ > 15%, and Δ_+_ < 13%. The gain *K*_*p*_ is set to 1 due to the considerations in section “The Gain Factor (*K*_*p*_).” We also choose the two best-fitting models according to Figure 5. Therefore, Figure 8 shows how a linear regression can predict the observed values of four of the parameters of the P2DUZ model. Figure 9 applies the linear regression for the same parameters, as well as the third pole −1/*T*_*p*__3_, of the P3DUZ model. The thicker line fit represents the aggregated data over three participants. Individual regression fits (the thinner lines) did not seem to offer any information, even though proper significance tests have not been performed. Therefore, the coefficients are only reported for the aggregated data. The linear predictor would estimate the value of each parameter based on the relative change in the step size, or Δ. As an example, the estimated value of the third pole of the modeled output using P3DUZ, in response to a sudden tempo decrease of proportion Δ can be predicted from −1/*T*_*p*__3__–_, where *T*_*P*_3_−_ = α_*T*_*P*_3_−__ + β_*T*_*P*_3_−__Δ. The numerical values of the linear regression coefficients, α, and β, can be picked from Table 4 for both negative and positive step changes.

**TABLE 4:** Intercept (α) and slope (β) in the linear regression α+βΔ, for predicting P2DUZ and P3DUZ parameters including delay (*T*_*D*_), damping ratio (ζ), oscillation period (*T*_ω_), zero (−1/*T*_*Z*_), and the third pole (−1/*T*_*p*__3_) (for the P3DUZ model), for the supraliminal step sizes, negative or positive based on a tempo step’s relative jump from a baseline of 100 bpm.

Model	P2DUZ		P3DUZ	
Parameter	(α_–_, β_–_)	(α_+_, β_+_)	(α_–_, β_–_)	(α_+_, β_+_)
*K*_*p*_	(1, 0)	(1, 0)	(1, 0)	(1, 0)
*T*_*D*_	(0.1150, 0.0161)	(0.1281, −0.077)	(0.0296, 0.0403)	(0.0416, 0.0135)
ζ	(0.6865, −0.307)	(0.7303, 0.3830)	(0.7500, −0.422)	(0.6114, 0.6903)
*T*_ω_	(0.3416, −0.168)	(0.3649, −0.271)	(0.2563, −0.112)	(0.3093, −0.458)
*Tz*	(0.8466, −0.727)	(1.0063, −0.375)	(0.7663, −0.025)	(1.2166, −0.655)
*T*_*p*__3_	(0,0)	(0,0)	(0.395, 0.6563)	(0.3272, 0.0566)

## Conclusion

Three human subjects participated in an SMS experiment where each participant was presented with a randomized set of 27 sequences of click sounds that started from a 100-bpm base tempo. The tempo was then changed in steps to and from a range of 102–200 bpm, back and forth. All participants’ tap responses were aggregated over 60–240 repetitions for each tempo step size. The discrete tap-events were upsampled to a sampling frequency of 60 Hz, to give a sampled input stimulus interval signal and a representative output interval signal. Using models from control theory to study the period error correction process in this behavioral SMS task, we fed these signals to a system identification algorithm. We argued that at a minimum, the inclusion of two complex poles, a delay, and one zero is necessary to capture the qualitative features of inter-response intervals, while an additional third pole can improve the model performance, in terms of least-square fit ratio, even further.

Results revealed a qualitatively distinct behavior under two regimes in agreement with the SMS literature. For the subliminal step-changes below the tempo change’s conscious awareness, despite the noisy measurements, a slow, gradual adaptation to the new rhythm was observed with an absent or a minimal overshoot. For larger step-changes, supraliminal steps, there was a relatively faster adaptation with an overshoot, without a considerable following undershoot. For the latter range, the model parameters of a P3DUZ model were described as a function of the step-size function, using a linear regression. Very small differences could be observed for the three individuals, so general linear relationships were derived.

## Future Work

Due to the labor-intensive, repetitive, experimental effort for gathering data with little noise, that spanned a large number of steps, the inclusion of more than three participants was deemed unfeasible in the scope of this study. This issue could be addressed in future studies by including other methodologies that could collect data from a larger set of participants.

This study dealt with the tempo adaptation and the period correction process. Separate efforts should be made to analyze the properties of asynchrony by studying the phase error correction, the other SMS’s process. Therefore, a similar experimental setup could be studied with a potentially different theoretical framework addressing the synchronization of event variables instead of interval variables. The smaller the change in the tempo, the longer it will take for the asynchrony to accumulate for the participant to detect that there has been a change. Therefore, the study of properties of asynchrony is particularly crucial for the subliminal range where the synchronization mechanism can be different. Thus, the relative change will worsen, the smaller the step, below the critical tempo change threshold.

This study focused on a simple sensorimotor task of in-phase finger-tapping in one-to-one to an auditory sequence of clicks, and was primarily presented to detail the methodology. Other discrete SMS tasks of anti-phase tapping, off-phase tapping (2:1, 1:2, 1:3, etc.), used with other mediums of hand-clapping, hitting different weights against a pad or other musical instruments can be investigated. In addition to the study of the auditory channel, visual or tactile stimuli can also be incorporated. Other types of inputs such as ramp tempo changes could as well be used, as previously included in a pilot project leading to the current work (Forbord, 2010).

The gradual change of the model parameter values, as the tempo step size changed, might be used as a guide toward developing a complete non-linear model. Such a non-linear model could then possibly handle general input signals with a rhythmic content, rather than just tempo step changes.

Finally, the current black-box approach models the whole system from sensory to action circuits. More detailed processes underlying SMS can further be investigated by breaking the system into smaller parts, such as an internal timekeeper, musculoskeletal system, and a controller inspired by Bégel et al. (2017). Penetrative methods such as EEG or fMRI in collecting intermediate signals can help revealing the transfer function for sensory, processing and motor subsystems separately to reveal each of these block’s transfer function. This requires accurate measurement and more advanced experimental setups with penetrative measurements within the sensorimotor circuitry.

Further studies that can use a similar methodology with other SMS tasks, or musically expressive Also, follow-up studies could possibly refine the methodology to make it less labor-intense. Although we have investigated the dependency of our parametrization on the size of the step change linearly, more data could reveal higher-order terms works.

## Data Availability Statement

The raw data supporting the conclusions of this article will be made available by the authors, without undue reservation.

## Ethics Statement

The studies involving human participants were reviewed and approved by Q2S Centre of Excellence, NTNU. Written informed consent for participation was not required for this study in accordance with the national legislation and the institutional requirements.

## Author Contributions

ND performed the experiments, modeling, and programming under US close supervision, which included original guidance, detailed discussions, and numerous proof-reading. Both authors contributed to the article and approved the submitted version.

## Conflict of Interest

The authors declare that the research was conducted in the absence of any commercial or financial relationships that could be construed as a potential conflict of interest.

## Footnotes

1 Our upsampled signals are still discrete-time signals, but the sampling frequency is high enough that a continuous-time analysis can be used.

2 Such as a participant at a sensorimotor task.

3 Superposition properties imply that the net response caused by two or more stimuli is the sum of the responses that would have been caused by each stimulus individually.

4 https://www.mathworks.com/help/matlab/ref/pchip.html

5 Prediction-error minimization https://ww2.mathworks.cn/help/ident/ref/pem.html

6 In the previous study, missed taps or claps were treated differently by inserting a hypothetical tap, i.e., dividing the IRI by two or a higher integer, if one or more onsets were missed in a trial.

7 The expected value here is 0.3% since in a normal distribution 99.7% of observations are supposed to fit within three standard deviations.

8 These are functions of irregular time points and can be upsampled/interpolated to be functions of regular time sample points, as described in the next section.

9 This will translate to a setting of kind = previous in staircase interpolation functions which simply return the previous value of the point (the initial ISI) until the new stimulus arrives as seen in the negative step of the Figure 3.

10 Minimizing the sum of squared residuals in the comparison between the modeled and the observed signals.

11 Note that as mentioned in 3.7, before feeding the signals to the system identification toolbox, we offset the output by adding an additional processing delay. Hence, this value should be subtracted from the estimated value of the *T*_*D*_.

12 Allowing a system to oscillate in pem function is simply achieved by including the letter U (for underdamped) in the name of the model.

13 Due to the higher ratio of jitter and other noise compared to the step size (section “Experimental Setup”) while normalizing all steps to the same value (section “Normalization”).

14 *Transcranial magnetic stimulation* (*TMS*) is a non-invasive method used to stimulate small regions of the brain.

15 One could explain that the gain factor estimate differs from 1 by suggesting that it may take longer than the participants’ available time series to settle to the final tempo.

16 This will result in a lower model performance for P2DU compared P2DUZ and P3DUZ for two reasons: first due to larger residuals where an undershoot is wrongfully modeled when the output has seemingly reached the input. Second, because the model has to underestimate the actual overshoot, as well as the estimated delay, to maximize the fit.

17 Including a third pole without a zero can also capture this feature but, based on the study of the fit ratio and the large noise in the performance of P3DU model in the toolbox which was observed during the preparatory stages of this work, we chose to not include a pole before a zero was introduced.
